# Did a quality improvement collaborative make stroke care better? A cluster randomized trial

**DOI:** 10.1186/1748-5908-9-40

**Published:** 2014-04-01

**Authors:** Maxine Power, Pippa J Tyrrell, Anthony G Rudd, Mary P Tully, David Dalton, Martin Marshall, Ian Chappell, Delphine Corgié, Don Goldmann, Dale Webb, Mary Dixon-Woods, Gareth Parry

**Affiliations:** 1 Director of Innovation and Improvement Science, Salford Royal NHS Foundation Trust, Stott Lane, Salford, M6 8HD, England; 2 Salford Royal Foundation Trust, Stott Lane, Salford, M6 8HD, England; 3 University of Manchester, Oxford Road, Manchester, M13 9PL, England; 4 Kings College London, 9th Floor, North Wing, St Thomas’ Hospital, Lambeth Palace Road, London, SE1 7EH, London; 5 Institute of Epidemiology & Health, Faculty of Population Health Sciences, University College London, Gower Street, London, WC1E 6BT, London; 6 Institute for Healthcare Improvement, 20 University Road, 7th Floor, Cambridge, MA 02138, UK; 7 Stroke Association, Stroke Association House, 240 City Road, London, EC1V 2PR, London; 8 Department of Health Sciences, University of Leicester, Leicester, LE1 7RH, England; 9 Harvard Medical School, Boston, MA 02115, USA

**Keywords:** Stroke, Quality improvement, Breakthrough series collaborative, Trial

## Abstract

**Background:**

Stroke can result in death and long-term disability. Fast and high-quality care can reduce the impact of stroke, but UK national audit data has demonstrated variability in compliance with recommended processes of care. Though quality improvement collaboratives (QICs) are widely used, whether a QIC could improve reliability of stroke care was unknown.

**Methods:**

Twenty-four NHS hospitals in the Northwest of England were randomly allocated to participate either in Stroke 90:10, a QIC based on the Breakthrough Series (BTS) model, or to a control group giving normal care. The QIC focused on nine processes of quality care for stroke already used in the national stroke audit. The nine processes were grouped into two distinct care bundles: one relating to early hours care and one relating to rehabilitation following stroke. Using an interrupted time series design and difference-in-difference analysis, we aimed to determine whether hospitals participating in the QIC improved more than the control group on bundle compliance.

**Results:**

Data were available from nine interventions (3,533 patients) and nine control hospitals (3,059 patients). Hospitals in the QIC showed a modest improvement from baseline in the odds of average compliance equivalent to a relative improvement of 10.9% (95% CI 1.3%, 20.6%) in the Early Hours Bundle and 11.2% (95% CI 1.4%, 21.5%) in the Rehabilitation Bundle. Secondary analysis suggested that some specific processes were more sensitive to an intervention effect.

**Conclusions:**

Some aspects of stroke care improved during the QIC, but the effects of the QIC were modest and further improvement is needed. The extent to which a BTS QIC can improve quality of stroke care remains uncertain. Some aspects of care may respond better to collaboratives than others.

**Trial registration:**

ISRCTN13893902.

## Background

Quality improvement collaboratives (QICs) bring together multidisciplinary teams across different organizations to work over a period of time towards defined improvement goals [1]. Though QICs have enjoyed enduring popularity as a means of securing improvement, evidence to support their effectiveness is equivocal [2]. This may be because studies of collaboratives have often used weak designs that are biased in favor of positive findings, tend towards measurement error, and perform poorly in ruling out alternative explanations for any improvement detected, including secular trends [3,4]. Further, collaboratives may tackle complex, multi-faceted interventions, some of which may have weak underlying evidence for their effectiveness [5]. In this article, we report a cluster-randomized trial of a QIC based on the Breakthrough Series (BTS) model that sought to improve compliance with processes associated with high-quality stroke care in hospitals in the Northwest of England.

Stroke affects up to 15 million people per year worldwide [6]. Of these, 17% die within one month. Survivors often suffer long-term disability, resulting in increased health and social care costs. The impact of stroke can be substantially mitigated by optimal care, but data from the 1990s showed that UK patients had poorer outcomes than many European countries [7]. For the last decade, the English National Sentinel Audit of Stroke (hereafter ‘National Audit’) has collected data biennially from hospitals in England and Wales on over two hundred measures of stroke care [8]. This has revealed dramatic variation in the care provided by different organizations, and has identified nine evidence-based processes associated with improved outcomes [9]. In 2008, prior to the commencement of this research, National Audit data indicated that stroke care in the Northwest of England was among the worst in the UK; for example, only four of every ten patients received a brain scan within 24 hours.

Stroke 90:10 was a BTS collaborative that aimed to improve compliance to an average of 90% on each of the nine processes by the next National Audit (2010), up from the baseline average of 72% in 2008. The nine processes targeted by the collaborative were in effect pre-defined, since they had already been selected as the basis of performance assessment for the National Audit. However, the aims of Stroke 90:10 went beyond simply enhancing reported performance on the national measures*.* The QIC sought to improve not just average compliance, but also reliability. Reliability was defined to mean reducing variation and delivering higher levels of consistent performance. To motivate and assess reliability, the nine key processes were organized into two care bundles: one for early hours care (Bundle 1) and one for rehabilitation (Bundle 2) [10].

We conducted a cluster randomized controlled trial with an interrupted time series design to determine whether hospitals participating in the Stroke 90:10 collaborative improved more than non-participating controls, as assessed by compliance with the two bundles of care. We compared across collaborative and control hospitals the amount of improvement on bundle compliance.

### Quality improvement collaboratives

A QIC is an organized, multifaceted approach to quality improvement that involves five essential features: an agreed topic and aim; clinical and quality improvement experts who provide support for improvement by acting as a faculty; multi-professional teams from multiple sites who participate in the QIC; use of an agreed model for improvement (for example, setting an aim, collecting data and testing changes); and the collaborative process involves a series of structured activities including face-to face-meetings [5].

As well as mastering quality improvement techniques and applying them in their own organizations, teams participating in QICs are encouraged to create a sense of common purpose and commitment, to generate enthusiasm, mutual support, and shared learning [11]. Several variants of QIC models exist, with the Institute of Healthcare Improvement’s BTS design among the most widely used [1]. A BTS collaborative typically involves collaborative faculty comprising quality improvement experts, three learning sessions (collaborative meetings), and action periods where participants implement activities, including Plan-Do-Study Act (PDSA) cycles. QICs are often used to implement evidence-based processes grouped into care ‘bundles’ or composites of processes. Behind the concept of bundles is the idea that groups of interrelated processes, rather than individual processes, are the appropriate target both for standards of practice and for analysis [12]. Bundles require that all patients receive all elements of the bundle (or have documented evidence of valid exception). There is also a theory that the bundle as a whole will achieve better results than the sum of its parts [13].

### The stroke 90:10 QIC

The Stroke 90:10 collaborative ran from July 2008 to December 2010, with participating organizations recruited between July and December 2008. Participating site teams received an extensive package of support from the program office, which was based at Salford Royal Hospitals NHS Trust. Support included executive mentoring visits; direct access to the Stroke 90:10 project director; an improvement advisor and one another via a web-based portal (extranet); and weekly online sharing and learning sessions. Teams were asked to produce monthly reports to reflect on their performance. The project director (author MP) also met with each hospital’s chief executive and the team to review progress twice.

All hospitals participating in the collaborative were asked to commit to:

1. appointing an executive lead, a physician leader, a site lead, and a project team comprising relevant leaders from clinical and ward areas;

2. taking part in one two-day and two one-day learning sessions, 90 days apart, that provided instruction in the theory and practice of improvement, offered teams advice and guidance, and shared cumulative results;

3. participating in ongoing collaborative activities over the course of the program;

4. using ‘The Model for Improvement’ to implement changes at the point of care and test them for local feasibility, reliability, and evidence of improvement in relation to the two bundles of processes relating to stroke care (Table 1) [14];

5. collecting data on 20 randomly selected patients per month;

6. submitting the data to a bespoke web-based system linked to the National Audit.

**Table 1:** Care bundles for stroke 90:10

	
**Early hours (Bundle 1)**	1. **Brain imaging** within 24 hours of admission to hospital (CT scan) to confirm stroke type (ischaemic or haemorrhagic) and determine management.
	2. **Delivery of aspirin or an alternative antiplatelet** (for patients where an antiplatelet is clinically indicated) within 24 hours of admission to modulate stroke complications and improve outcomes. For shorthand, we refer to this as ‘aspirin.’
	3. **Swallow screen** within 24 hours of admission, to prevent unnecessary withdrawal of nutrition, support timely administration or modification of aspirin/antiplatelet delivery and highlight patients who need on-going management of swallow safety.
	4. **Weight assessment** on admission, as a marker of the likelihood of repeated weighing and diligent management of nutrition.
**Rehabilitation (Bundle 2)**	1. **Physiotherapy assessment** within 72 hours of admission to improve early mobilization, and increased likelihood of targeted goal setting.
	2. **Occupational therapy assessment** within 4 days of admission to support activities of daily living, memory, perception and cognition.
	3. **Mood assessment** (during the in-patient stay) to screen for altered mood and other factors, given that post-stroke depression is known to affect the likelihood of long-term recovery.
	4. **Documented evidence of MDT goals set for rehabilitation** as a marker of patient involvement in care and multidisciplinary team working.
	5. **50% of the patient’s hospital stay on a stroke unit**, defined using the National Audit criteria, given evidence that stroke units reduce mortality and improve patient outcomes.

## Methods

### Study design

A cluster randomized controlled trial was used with an interrupted time series design to compare the hospitals participating in the Stroke 90:10 QIC with the non-participating controls. The study sought to compare the amount of improvement on the two evidence-based bundles of care (early hours and rehabilitation) used in the QIC.

This study was approved by Tameside and Glossop Research Ethics Committee (Ref: 08/H1013/55) and was registered as a clinical trial with the International Standard Randomized Controlled Trial Number Register (Ref: ISRCTN13893902).

### Setting and participants

All NHS hospital Trusts in the Northwest of England were invited to participate based on the pre-defined inclusion criteria of: a minimum of ten inpatient dedicated stroke beds (a ‘stroke unit’); agreement to participate signed by the chief executive; agreement to participate from a consultant in stroke medicine (or equivalent); a dedicated multidisciplinary stroke team; and availability of case notes for review.

### Randomization

We used a stratified-randomization approach. Hospitals were stratified by stroke performance (Sentinel Audit score above or below 60) in the 12 months preceding baseline data collection (2007 and 2008). Within each group, a computer-generated list was used to randomly allocate 12 hospitals to the intervention group and 12 to the control group. The intervention group took part in the QIC from January to October 2009, but the control group did not join the QIC until January 2010. The control group of hospitals, who waited to join the QIC, provided an ‘untreated’ control group. The nature of the trial meant that participants could not be blinded to group allocation.

#### Sample size

A conservative approach to the power calculation was undertaken, based on a simple baseline average versus end of study average comparison. This was preferable to basing the power calculation on an interrupted time series using a fixed number of charts per month, because we did not know in advance how the sites would choose to implement their activities or the likely impact of these activities (for example, in a simple linear fashion, stepped or incremental, or any other way).

Various permutations of the power calculation were performed. Using data from the 2008 National Stroke Audit, the intra-class correlation coefficient was estimated at 0.149 for Bundle 1 and 0.217 for Bundle 2. Based on preliminary data, where at one hospital, compliance with Bundle 2 had improved to 80%, we assumed a 0.05 significance level, 90% power and a minimum detectable difference of 25% for Bundle 1 and 35% for Bundle 2 in relative increased uptake between the control and intervention. We estimated that for improvement on Bundle 1 to reach significance, up to 24 hospitals, with 12 on either arm, would be required (based on the control group improving to 40% compliance and the intervention group improving to 65% compliance). For Bundle 2 to reach significance, up to 19 hospitals (10 on either arm) would be required (based on the control group improving to 45% compliance and the intervention group improving to 70% compliance).

### Outcomes and follow-up

Participating hospitals provided data to the QIC separately and independently from the National Audit (Table 2), although both were measuring the same processes. Random samples of 20 patients per month per hospital were used to generate data for both the intervention period (July 2008 to December 2009) and baseline pre-intervention period (July 2008 to December 2008).

**Table 2:** Baseline information for individual data (patient level)

	**Intervention**		**Control**		**P**
**Male**	2,287	48.2%	2,502	47.3%	0.559
**Comorbidities**					
Atrial fibrillation	714	15.1%	768	14.5%	0.457
Previous stroke or TIA	1,299	27.4%	1,404	26.6%	0.328
Diabetes mellitus	735	15.5%	742	14.0%	0.178
Hyperlipidaemia	647	13.6%	735	13.9%	0.942
Hypertension	2,087	44.0%	2,122	40.1%	0.135
Myocardial infarction or angina	777	16.4%	705	13.3%	0.019
Valvular heart disease	150	3.2%	118	2.2%	0.188
Cardiac failure	113	2.4%	135	2.6%	0.899
**Risk factors**					
Smoker	806	17.0%	765	14.5%	0.064
Ex smoker	733	15.5%	903	17.1%	0.453
Alcohol excess	275	5.8%	304	5.7%	0.692
Patient independent pre stroke	3,285	69.3%	4,031	76.2%	0.384
Readmitted within 28 days	356	7.5%	308	5.8%	0.223
Patient died as inpatient	1,006	21.2%	1,011	19.1%	0.189
Alive at 30 days	3,646	76.9%	3,834	72.5%	0.835
Patient received antibiotics for a newly acquired pneumonia during admission after stroke	298	6.3%	298	5.6%	0.729
Patient had urinary tract infection in first seven days of admission defined by a positive culture or clinically treated	159	3.4%	144	2.7%	0.514
Motor deficits in first 24 hrs of admission	2,534	53.4%	2,795	52.9%	0.727
Dysphasia in first 24 hrs of admission	1,588	33.5%	1,753	33.2%	0.748
Dysarthria in first 24 hrs of admission	1,549	32.7%	1,526	28.9%	0.808
Motor deficits in first 24 hrs of admission	2,534	53.4%	2,795	52.9%	0.879
Patient worst level of consciousness					0.992
Unconscious	527	11.1%	507	9.6%	
Confused	264	5.6%	248	4.7%	
Drowsy	657	13.9%	884	16.7%	
Fully conscious	2,933	61.9%	3,575	67.6%	
Not known	360	7.6%	73	1.4%	

**Figure 1:**
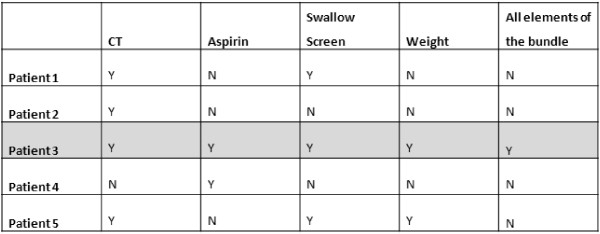
**Bundle compliance.** In this example only patient 3 would be classed as receiving care that was bundle compliant.

The primary measures for the trial were compliance with Bundle 1 and Bundle 2. The elements measured within those bundles were identical to the indicators used for the National Audit [8]. Bundle compliance was calculated using an ‘all or none’ measurement, where every process element of the bundle had to be undertaken for overall bundle compliance to be achieved [15]. Thus, omission of any one or more elements would result in a patient’s care being deemed ‘bundle non-compliant’ as illustrated in Figure 1.

### Data collection

Once the QIC began in January 2009, intervention teams were asked to submit, every month, a complete registry of discharged patients coded for stroke from the previous month (based on ICD 10 codes 61, 63, and 64). Each month, the Stroke 90:10 faculty used this registry to produce the random sample of 20 patients for each hospital to audit. For each of the 20 patients included in the sample, data were collected on 45 questions (excluding basic demographic information) on compliance with elements of Bundle 1 and Bundle 2, the three stages of acute stroke care: stroke onset and hospital stay; comorbidities and risk factors; and standards by discharge. Data were entered into a web-based database developed in partnership with the Royal College of Physicians.

The QIC faculty collected the data for the baseline period for hospital teams in the intervention group, and throughout the entire study period for those in the control group. After the baseline period the intervention group were expected to collect and submit data themselves, though they sometimes required extensive faculty support to do so.

**Figure 2:**
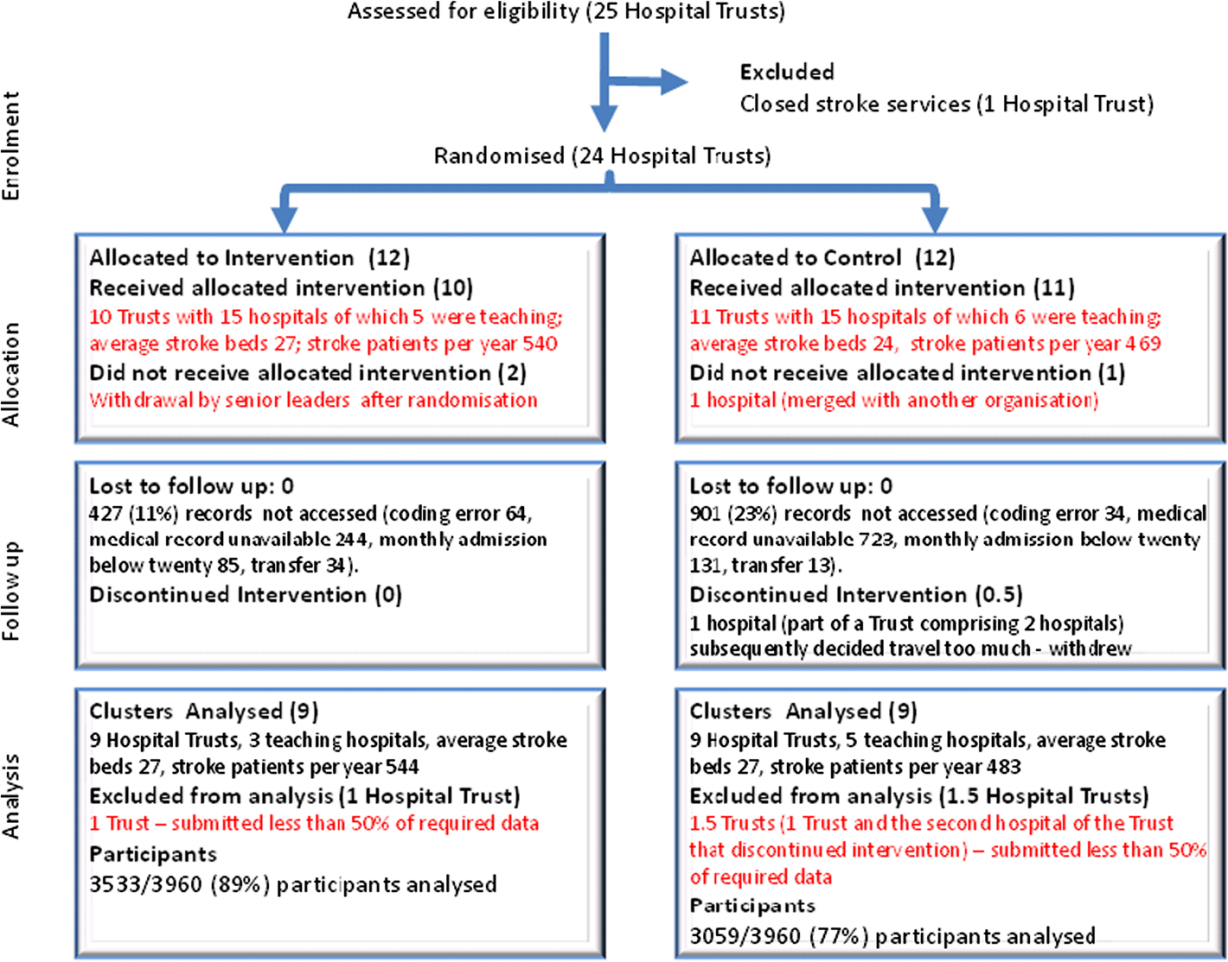
Flow diagram of participation.

In both the control and intervention groups, hospitals were asked to audit all patients in months where there were fewer than 20 admissions. Where patient records were incomplete, could not be retrieved, or were miscoded, patients were excluded and replaced by the next available patient. In total, 1,328 (17%) records (control and intervention) were excluded (see Figure 2).

### Statistical analysis

We used a difference-in-difference approach to compare the differences between the intervention and control groups on bundle compliance. This approach measures the difference in bundle compliance over time (before and after the intervention) for the intervention group compared with the difference over the same period for the control group. The analysis was carried out using STATA (version 11, StataCorp LP, College Station, TX, USA).

Using a pre-post-test methodology, we aimed to determine whether there was a difference in relative average bundle compliance in the last three months of the baseline period (October 2008 to December 2008) compared with the last three months of the collaborative (October 2009 to December 2009). Specifically, we sought to determine a difference in difference of means. We fitted a logistic regression model with a random effects term to take account of clustering at the hospital level. Bundle compliance was the primary measure. Explanatory variables included group (control or intervention), time period (October 2008 to December 2008 and October 2009 to December 2009) and an interaction term between them. The interaction term provides the difference in differences that estimates the impact of the collaborative.

## Results

Of the 25 eligible trusts in the Northwest of England, 24 (covering 30 hospitals) agreed to participate and were randomized (Additional file 1). Of these, data were available from 18 trusts for analysis (Figure 2), yielding 3533 patients in the intervention arm and 3,059 patients in the control arm. Comparisons of patient demographic and clinical data between the control and intervention groups (Table 2) suggested no significant differences at baseline, except that the intervention group patients showed higher co-morbidity from myocardial infarction or angina (16.4% versus 13.3%; p = 0.019). The collaborative program was run as designed. However, hospital sites did not consistently audit 20, or all, patients each month. Figure 2 shows that a small number of hospitals were excluded for having a reporting rate under 50%; this was pre-specified in the protocol.

### Primary measures

#### Bundle 1 (early hours)

**Figure 3:**
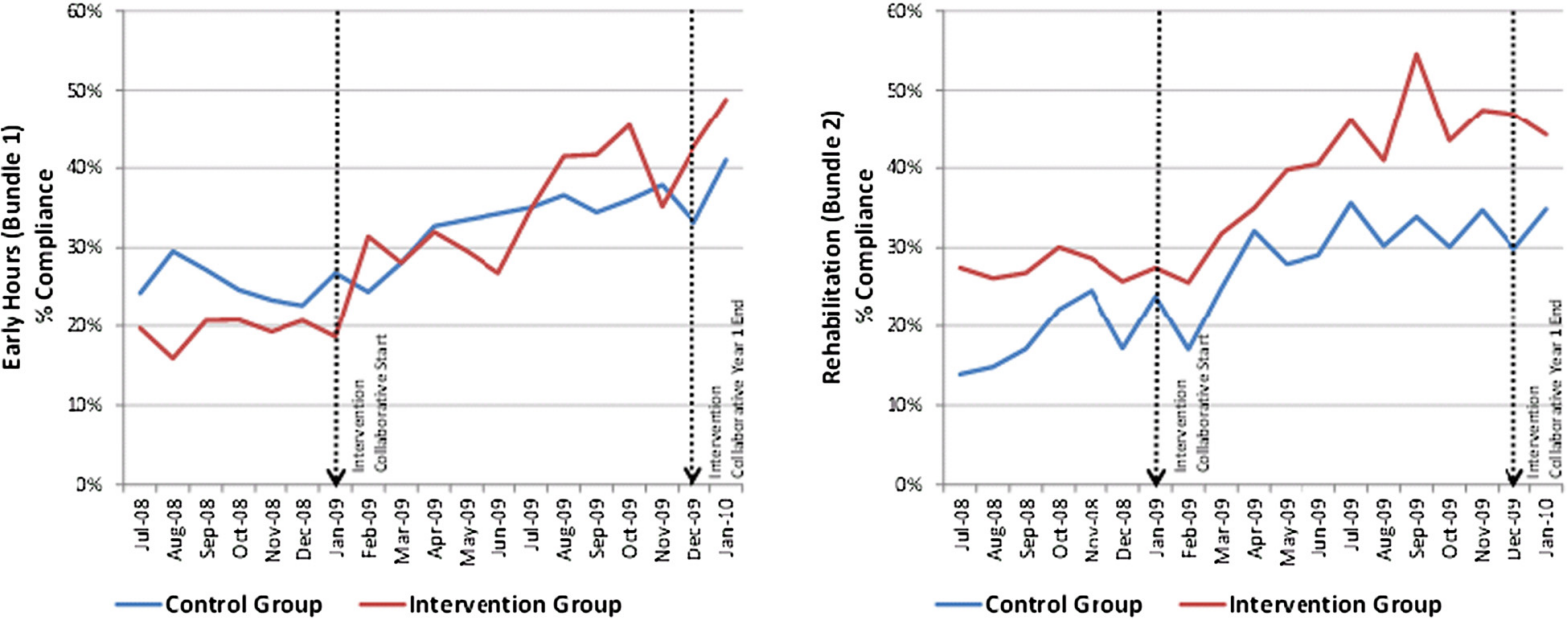
**Bundle compliance over time in the control and intervention group.** In bundle 1, for the control group, compliance went from 24.3% to 37.5% (13.2% change) and in the intervention group, from 19.6% to 42.3% (22.7% change). On crude comparison, this is a difference of 9.5%, but with all the various adjustments for clustering, this results in a relative benefit of 10.9% in the intervention relative to the control group.

Average Bundle 1 compliance in the control group at baseline (October 2008 to December 2008) was 24.3%, rising to 37.5% by study end (Figure 3). In the intervention group, compliance was 19.6% at baseline, rising to 42.3% by study end. Using a difference-in-difference approach, this represents a relative increase in the odds of compliance of 1.56 (95% CI 1.06, 2.31 p = 0.025) in the intervention group compared with the control group (Table 3). These odds are equivalent to a relative improvement by the end of the study of 10.9% (95% CI 1.3%, 20.6%) in the intervention group relative to the control group.

**Table 3:** The proportion of patients who received the bundle in the three months prior to the start of the collaborative intervention (October 2008 to December 2008) were compared with the proportion who received the bundle in the three months at end of the intervention (October 2009 to December 2009)

	**Compliance: Proportion (%)**				**Difference in differences of averages**		
	**Control group**		**Intervention group**				
	**Oct-Dec 2008**	**Oct-Dec 2009**	**Oct-Dec 2008**	**Oct-Dec 2009**	**Odds ratio 95% CI Percent relative improvement**	**P**	ICC*
**Early hours (Bundle 1)**	122/502	212/566	114/583	258/610	1.56 (1.06, 2.31)	0.025	0.066
	24.3%	37.5%	19.6%	42.3%	10.9% (1.3%, 20.6%)		
Brain Scan within 24 hrs of Hospital Admission	316/481	435/556	353/568	455/603	0.95 (0.65, 1.4)	0.799	0.064
	65.7%	78.2%	62.1%	75.5%	-0.9% (-8.6%,5.4%)		
Aspirin in 24 hrs of hospital Admission	151/392	258/463	115/436	261/505	1.44 (0.96, 2.15)	0.079	0.051
	38.5%	55.7%	26.4%	51.7%	9% (-1%,18.8%)		
Swallowing Screening Recorded in 24 hrs of Hospital Admission	220/422	350/500	326/495	426/523	1.09 (0.71, 1.67)	0.7	0.162
	52.1%	70%	65.9%	81.5%	2% (-8.4%,11.2%)		
Weighed during Hospital Admission	258/415	346/504	286/491	462/525	4.4 (2.75, 7.03)	<0.001	0.158
	62.2%	68.7%	58.2%	88%	26.2% (20.1%,30.6%)		
**Rehabilitation (Bundle 2)**	110/502	188/566	159/583	282/610	1.61 (1.07, 2.42)	0.023	0.197
	21.9%	33.2%	27.3%	46.2%	11.2% (1.4%,21.5%)		
Ward of 50 percent + of stay	325/502	414/566	384/583	473/610	1.17 (0.8, 1.72)	0.412	0.091
	64.7%	73.1%	65.9%	77.5%	3.0% (-4.6%,9.3%)		
Physiotherapist assessment in 72 hrs of Hospital Admission	325/502	414/566	384/583	473/610	1.6 (0.98, 2.6)	0.06	0.119
	64.7%	73.1%	65.9%	77.5%	9% (-0.4%,16.1%)		
Occupational Therapist Assessment in 4 days of Hospital Admission	302/406	393/495	394/490	479/534	1.06 (0.68, 1.67)	0.789	0.234
	74.4%	79.4%	80.4%	89.7%	1.4% (-9.5%,11.1%)		
Mood Assessed during Hospital Admission	235/388	354/478	330/469	419/515	2.68 (1.69, 4.26)	<0.001	0.247
	60.6%	74.1%	70.4%	81.4%	24.1% (12.9%,34%)		
Rehabilitation Goals set during Hospital Admission agreed by MDT	147/407	226/488	192/478	315/512	5.43 (3.26, 9.05)	<0.001	0.194
	36.1%	46.3%	40.2%	61.5%	35.8% (27.3%,41.9%)		

*ICC: The Intraclass Correlation Coefficient indicates the variation due to patients being clustered within hospitals. The *ICC* estimates shown here are derived from the associated random effects model.

In line one the numerator is the number of patients who received all four process measures; the denominator is the total number surveyed in the three month period.

Secondary analysis found, within the bundle processes, that the largest relative difference was seen in administering aspirin (or an alternative antiplatelet) within 24 hours of admission. The odds of aspirin (or suitable alternative) administration to patients where it was clinically indicated in the intervention relative to the control group increased by 1.52 (95% CI 1.05, 2.2 p = 0.027).

#### Bundle 2 (rehabilitation bundle)

Baseline bundle compliance with Bundle 2 in the control group was 21.9%, rising to 33.2% by study end. In the intervention group, baseline compliance was 27.3% and rose to 46.2% by study end (Figure 3). Using a difference-in-difference approach, this represents an increase in the odds of compliance of 1.61 (95% CI 1.07, 2.42 p = 0.023) in the intervention group compared with the control group. These odds are equivalent to a relative improvement by the end of the study of 11.2% (95% CI 1.4%, 21.5%) in the intervention group relative to the control group (Table 3).

Secondary analysis found that for the individual processes included in the bundle, both Mood Assessed during Hospital Admission (odds 1.74, 95% CI 1.17, 2.58; p = 0.006) and Rehabilitation Goals set by multidisciplinary teams MDT (odds 2.43, 95% CI 1.7, 3.47; p < 0.001) increased in the intervention group more than in the control group.

## Discussion

Our study is among a small number of studies evaluating the effectiveness of a QIC that have used a controlled design. Like others [9,16], we have found evidence of improvement in both the intervention and control groups pointing to an underlying secular trend of incremental improvement over time. Some of the reasons for this are likely to lie with the unprecedented national and regional attention on stroke coinciding with the period of study. During this time, managed clinical networks for stroke were being developed, a National Audit Office report was published and the Department of Health Stroke Improvement program was launched [17]. Delivery of thrombolysis was a national target, and the basic processes required for success in achieving this were closely linked to the processes of care packaged into our Early Hours bundle. Given the intensity of focus, it is unsurprising that both intervention and control groups demonstrated improved performance. The trends we observed are significant and signal the collective impact of direction setting (through national policy), implementation of national standards, regulation, clinical leadership, research implementation and other activities in creating a ‘rising tide’ of more reliable care for stroke patients.

Participation in a QIC does appear to offer a modest overall effect in the amplitude of change over and above secular trend. At first glance it may seem disappointing that the overall benefits are not more significant. However, the bundled nature of our primary outcome measures may have obscured some important and significant effects. These include the disproportionate impact of Stroke 90:10 on some process indicators of quality of care and the resistant nature of others. Looking at the bundles of care as individual items, it is possible to see that four care processes (administration of aspirin/antiplatelet, weighing patients on time, mood assessment and setting MDT rehabilitation goals) improved significantly in the group of hospitals that participated in the collaborative. Weighing patients and setting MDT goals conferred a 17% and 21% absolute improvement respectively. One possibility, therefore, is that the effects of QICs may be specific rather than generalized: some processes of care may be more susceptible to the improvement methods used in a collaborative program than others. This is an important new hypothesis that requires further testing. Furthermore, it appears that these processes could have some distinctive characteristics: they are (relatively) simple changes under the direct control of the hospital stroke team on the stroke unit (defined as a physical unit and not a collection of staff and services) and they are geographically bounded. This possible explanation is supported somewhat by an Australian study in acute stroke units, which showed that focused improvements in direct patient care, delivered on a ward, can be made using a collaborative method [18].

Processes of care that are less tractable may require more attention to engineering systems and processes. For example, delivering a CT scan within hours of admission requires coordination of multiple services and provider groups. Achieving the necessary changes in patterns of working, culture, and attitudes requires an approach different from that used for less socially complex problems. Our study may thus provide some evidence to suggest that the effects of QICs, rather than being uniform, may be mediated by the nature of the processes being targeted and the amount of control participants have over them. Collaboratives may be more suitable for some kinds of improvement interventions than others, and future research should investigate this possibility further. The kinds of implementation interventions needed to support more complex organizational change also require further study, now that promising examples are beginning to emerge [19-24].

### Limitations

Though our study design represents improvement on the weaker designs typically used in evaluations of collaboratives, it does have some limitations. First, because the nine processes used in the study were those used by the National Audit, we may not have captured other processes of care that are clinically more important. Variation in data collection approaches across hospitals, although small, may have introduced bias to the results. Second, six hospitals did not provide data, and there was some variation in completeness rates between hospitals. This may introduce bias because it is unlikely the hospitals that failed to provide data, or did not produce complete data, were similar to those that did. Third, generalizability beyond an English context is limited. The socio-technical nature of this intervention, and the context within which it was set, suggests that it is not clear whether the same effects would be found in other populations or settings.

Fourth, our study was not designed to explain whether the increased process adherence resulted in improved outcomes or had any unintended consequences, for example longer lengths of stay or increased re-admissions to hospital. Future studies should investigate the possibility that there may be some causal mechanism that links improved performance on the nine processes to beneficial or unwanted outcomes. Finally, beyond evaluations of collaboratives, experimental evaluations of many complex socio-technical interventions demonstrate modest effect sizes, which might suggest the limitations of positivist evaluations in this context and signal a requirement for more sophisticated evaluation approaches. Some of these issues are the subject of an accompanying paper [25].

## Conclusions

Our study suggests that the answer to whether a Breakthrough Series QIC can deliver the extra boost needed to induce improvement beyond secular trend is not straightforward. It does appear to support improvement in more consistent delivery of some processes of care grouped into bundles, but additional, or other kinds of, support may be needed for more complex organizational challenges. Our study reinforces the need, when researching health service improvements, for controlled studies using difference-in-difference analyses to avoid mistaking secular trends for treatment effects. Delivering consistently high quality of stroke care remains a key challenge.

### Ethical approval

This study was approved by Tameside & Glossop Local Research Ethics Committee (Ref: 08/H1013/55).

## Competing interests

All authors have completed the Unified Competing Interest form at http://www.icmje.org/coi_disclosure.pdf (available on request from the corresponding author) and declare: DC, MPT and IC’s work on Stroke 90:10 was funded by The Health Foundation (MPT was subcontracted from Salford Royal NHS Foundation Trust and IC and DC continue to be employees of Salford Royal NHS Foundation Trust); PJT is Clinical Lead, Greater Manchester Stroke network and Clinical Lead Stroke Improvement National Audit Project. MPT is named as an applicant on a grant awarded as part of the Health Foundation’s Safer Clinical Systems program. MM and DW worked for the Health Foundation and contributed to the design of the study but had no undue influence over the data or interpretation. AR is Stroke Program Director at the Royal College of Physicians, responsible for the National Audit. No other relationships or activities that could appear to have influenced the submitted work are declared.

## Authors’ contributions

MP, PT, AR, MP, DG, DD and GP, with support from MM and DW made substantial contributions to the conception and design the study. MP, IC and CD undertook data collection. IC and GP undertook data analysis. All authors were responsible for interpretation of the analysis. MDW, MP, and GP drafted the paper and all authors critically reviewed, read and approved the final manuscript.

## Supplementary Material

Additional file 1: Table S1 CONSORT 2010 checklist of information to include when reporting a cluster randomised trial. Click here for file
